# Multicellular Complex Tumor Spheroid Response to DNA Repair Inhibitors in Combination with DNA-damaging Drugs

**DOI:** 10.1158/2767-9764.CRC-23-0193

**Published:** 2023-08-25

**Authors:** Thomas S. Dexheimer, Nathan P. Coussens, Thomas Silvers, John Wright, Joel Morris, James H. Doroshow, Beverly A. Teicher

**Affiliations:** 1 Molecular Pharmacology Laboratories, Applied and Developmental Research Directorate, Frederick National Laboratory for Cancer Research, Frederick, Maryland.; 2 Division of Cancer Treatment and Diagnosis, NCI, Rockville, Maryland.

## Abstract

Multicellular spheroids comprised of malignant cells, endothelial cells, and mesenchymal stem cells served as an *in vitro* model of human solid tumors to investigate the potentiation of DNA-damaging drugs by pharmacologic modulation of DNA repair pathways. The DNA-damaging drugs, topotecan, trabectedin, and temozolomide were combined with varied inhibitors of DNA damage response enzymes including PARP (olaparib or talazoparib), ATM (ataxia telangiectasia mutated; AZD-1390), ATR (ataxia telangiectasia and Rad3-related protein; berzosertib or elimusertib), and DNA-PK (DNA-dependent protein kinase; nedisertib or VX-984). A range of clinically achievable concentrations were tested up to the clinical *C*_max_, if known. Mechanistically, the types of DNA damage induced by temozolomide, topotecan, and trabectedin are distinct, which was apparent from the response of spheroids to combinations with various DNA repair inhibitors. Although most combinations resulted in additive cytotoxicity, synergistic activity was observed for temozolomide combined with PARP inhibitors as well as combinations of the ATM inhibitor AZD-1390 with either topotecan or trabectedin. These findings might provide guidance for the selection of anticancer agent combinations worthy of further investigation.

**Significance:**

Clinical efficacy of DNA-damaging anticancer drugs can be influenced by the DNA damage response in tumor cells. The potentiation of DNA-damaging drugs by pharmacologic modulation of DNA repair pathways was assessed in multicellular tumor spheroids. Although most combinations demonstrated additive cytotoxicity, synergistic cytotoxicity was observed for several drug combinations.

## Introduction

The preservation of genomic integrity is essential for the maintenance of normal cellular functions, so multiple mechanisms have evolved to protect and repair DNA. Damage to DNA, induced by chemical or physical insults, is detected and subsequently repaired by robust and complex pathways (1). Defects to these pathways promote genomic instability, which is an enabling characteristic and hallmark of cancer that increases the acquisition of genetic variants to drive tumorigenesis (2–4). However, genomic instability can increase the sensitivity of cancer cells to DNA-damaging chemotherapies (5) and specific defects in genome maintenance pathways have been exploited by targeted synthetic lethal therapies (6).

Among the most toxic DNA lesions are double-strand breaks (DSB), where the sugar-phosphate backbones of both strands are broken within a sufficient proximity to cause a physical separation of the double helix (1). Following a DNA single-strand break (SSB), the intact complementary strand can serve as a template for repair. In contrast, DSBs are more difficult to repair and can cause fragmentation, chromosomal rearrangements, and a loss of genetic information and chromosomes (7). The PARP enzymes are involved in repairing DNA SSBs via base excision repair (BER) and DSBs through homologous recombination (HR) and non-homologous end joining (NHEJ; ref. 8). Inhibitors of PARP enzymes, such as talazoparib and olaparib, have been shown to prevent DNA repair by trapping the PARP protein at the site of DNA damage. The PARP inhibitor–PARP–DNA complex is lethal in HR-deficient cells such as those with alterations to *BRCA1/2* (9). Multiple DNA damage signaling pathways are facilitated by the phosphatidylinositol 3-kinase-like kinase (PIKK) family members: ATR (ataxia telangiectasia and Rad3-related protein), ATM (ataxia telangiectasia mutated), and DNA-PK (DNA-dependent protein kinase). ATR is recruited to single-strand DNA coated with replication protein A and is activated by single-strand–double-strand junctions that result during DNA repair and from the collapse of a replication fork (1). Because of the substantial replication stress of cancer cells, ATR is considered an attractive target for anticancer therapy (10, 11) and selective ATR inhibitors that are currently in clinical trials include berzosertib (M6620, VX-970) and elumusertib (BAY-1895344; Table 1). ATM is critical for the repair of DNA DSBs (12, 13) and is recruited to the site of DNA damage by the MRE11–RAD50–NBS1 (MRN) complex. ATM is activated at the MRN complex to initiate signaling pathways and influence whether the repair is made by HR or NHEJ (12). A deficiency in ATM or its low expression is a biomarker of sensitivity to PARP or ATR inhibitors in cancer (14–17) and the ATM-selective inhibitor AZD-1390 is currently in clinical trials (Table 1). DNA-PK is phosphorylated by ATM and is recruited to DNA DSBs by the Ku70–Ku80 complex where it initiates an extensive signaling cascade to facilitate religation through NHEJ (7, 12, 18). Inhibition of DNA-PK activity prevents the assembly of short-range complexes between the DNA ends (10, 19) and the DNA-PK selective inhibitors nedisertib (M3814) and VX-984 are currently under evaluation in clinical trials (Table 1).

**TABLE 1:** Drugs and investigational agents used in this study. The agents included DNA-damaging drugs (top) and DNA repair inhibitors (bottom). If available at the time of this study, the clinical *C*_max_ is listed. Also shown are the molecular target and current clinical status of each agent

DNA-damaging drugs	Clinical *C*_max_ (μmol/L)	Molecular target	FDA-approved indications and/or clinical status
Temozolomide	37.6	DNA	Glioblastoma multiforme; anaplastic astrocytoma (1999)
Topotecan	0.015	Topoisomerase 1	Ovarian; SCLC; cervical (1996)
Trabectedin	0.0024	DNA	Liposarcoma; leiomyosarcoma (2015)
**DNA repair inhibitors**	**Clinical *C*_max_ (μmol/L)**	**Molecular target**	**FDA-approved indications and/or clinical status**
Berzosertib (VX-970, M6620)	*NA*	ATR	Phase II
Elimusertib (BAY 1895344)	*NA*	ATR	Phase I
AZD-1390	*NA*	ATM	Phase I
Talazoparib	0.043	PARP	BRCA mutant, HER2^−^ breast (2018)
Olaparib	13.1	PARP	Ovarian; BRCA mutant, HER2^−^ breast, HR^+^ breast (2014)
Nedisertib (M3814)	*NA*	DNA-PK	Phase I/II
VX-984	*NA*	DNA-PK	Phase I

Abbreviation: *NA*, clinical *C*_max_ unknown.

Temozolomide, topotecan, and trabectedin are well-established anticancer drugs that damage DNA in different ways. Temozolomide is a prodrug that is spontaneously hydrolyzed at physiologic pH to form a short-lived species that alkylates the purine bases in DNA (20). Among the range of methyl adducts formed upon exposure to temozolomide, O^6^-methylguanine (O6MeG) accounts for a minor proportion but is the major cytotoxic lesion which severely impairs DNA replication due to the insertion of thymine opposite to methylguanine (21, 22). The O6MeG lesion can be resolved by DNA mismatch repair (MMR), BER, the alkylpurine-DNA-N-glycosylase (APNG) enzyme, or O^6^-methylguanine DNA methyltransferase (MGMT). In addition to MMR status, temozolomide sensitivity depends on the expression of MGMT, APNG, and BER proteins (22). Topotecan is an inhibitor of topoisomerase I, an enzyme which catalyzes the conversion of the DNA topology by introducing temporary SSBs. The formation of a ternary complex between topotecan, topoisomerase I, and DNA inhibits the religation of topoisomerase I-mediated DNA SSBs, which can eventually be converted into lethal DNA DSBs (23–25). The tetrahydroisoquinoline molecule trabectedin was originally isolated from the sea squirt (26). Trabectedin monoalkylates DNA at the exocyclic N2 amino group of guanine and binds tightly in the minor groove of the DNA double helix, which bends DNA toward the major groove (27). The cytotoxic activity of trabectedin has been linked to its interference with transcriptional regulation and transcription-coupled nucleotide excision repair, as well as the degradation of RNA polymerase II and the generation of DNA DSBs (26, 28). Sensitivity to trabectedin has been associated with deficiencies in HR (27–31). As monotherapies, each of these drugs had a sufficient efficacy to achieve FDA approval. However, their effects are diminished by cellular processes that rapidly repair drug-induced DNA lesions and enable tumor cell survival. Thus, combining a DNA repair inhibitor with temozolomide, topotecan, or trabectedin might enhance the tumor cytotoxicity if therapeutic efficacy is preserved. Although the inhibition of DNA damage repair pathways is a promising approach to improve the efficacy of DNA-damaging chemotherapies, caution is warranted because their systemic administration both potentiates the cytotoxicity of cancer cells and increases toxicity in normal tissues.

This study explores the anticancer activities of DNA-damaging drugs and inhibitors of DNA damage repair, both as single agents and in combinations (Table 1). Complex, multicellular tumor spheroids comprised of malignant cells, endothelial cells, and mesenchymal stem cells served as an *in vitro* model for human solid tumors (32). This model system incorporates physiologically relevant features and patient-derived or established malignant cell lines with clinically relevant genetic alterations from many tumor types. Twenty-six patient-derived or established malignant cell lines were evaluated representing sarcoma, melanoma, small cell lung cancer (SCLC), non–small cell lung cancer (NSCLC), as well as bladder, ovarian, and pancreatic cancers (Table 2). The concentration ranges selected for each drug and investigational agent were based upon clinically achievable concentrations if the clinical *C*_max_ of the agent was known.

**TABLE 2:** The malignant cell lines grown as complex tumor spheroids for this study. The names of both patient-derived and established cell lines are listed along with the tumor type they were derived from and key genetic alterations

Cell line	Tumor type	Genetic alterations
HS-SY-2	Synovial sarcoma	*SYT-SSX* fusion
SYO-1	Synovial sarcoma	*SYT-SSX* fusion, *BRCA2* G2044V
SW982	Synovial sarcoma	*BRAF* V600E, *PARP2* D235G
ASPS-1	Alveolar soft parts sarcoma	*ASPL-TFE3* fusion
VA-ES-BJ	Epithelioid sarcoma	*NF2* S87*
MPNST	Malignant peripheral neural sheath tumor	*NF1* R304*
287954-098-R-J1	Ewing sarcoma	*ARID1A* N2066Tfs*36, *TP53* R156P
NCI-H211	Small cell lung cancer	*TP53* R248Q
DMS 114	Small cell lung cancer	*TP53* R213*, *PARP9* R581G
NCI-H841	Small cell lung cancer	*TP53* C242S, *SMARXA4* K934*
SW 1271	Small cell lung cancer	*NRAS* Q61R, *BRCA1* K1233I
NCI-H1876	Small cell lung cancer	*NRAS* Q61K; *TP53* R273L
NCI-H196	Small cell lung cancer	*ATM* F582L, *BRCA2* V465V
COR-L88	Small cell lung cancer	*ATR* R1351L*, BRCA2* E443*, *TP53* V157F
NCI-H719	Small cell lung cancer	*TP53* R248Q
NCI-H1618	Small cell lung cancer	*PARP8* S121G*, PARP14* P998S*, RB1* P39Rfs*26
NCI-H226	Non–small cell lung cancer	*SMARCB1* low
NCI-H322M	Non–small cell lung cancer	*TP53* R248L, *ARID1B* low
349418-098-R	Non–small cell lung cancer	*BRAF* V600E*; ARID1A* S2249*
BL0293-F563	Bladder cancer	^a^ *BRCA2* R2034C; *ARID1A* S138*; *TP53* R248Q
556581-035-R-J1	Ovarian cancer	*PIK3CA* Q546R, *ARID1A* G1282*, ARID1B Y1346Ifs*102
G-401	Rhabdoid tumor	*SMARCB1* deletion
292921-168-R-J2	Pancreatic cancer	^a^ *KRAS* G12D, *GNAS* R201H, *RAD50* E723Gfs*5, *BRCA2* R3384*
156681-154-R-J1	Melanoma	*BRAF* V600E, *ARID1A* Y762_S763delins*, *CDKN2A* X153_splice
283228-195-R-J1	Melanoma	*NRAS* Q61R, *ATM* D1853V, ARID1A Q1200*
425362-245-T-J1	Melanoma	*BRAF* V600E, *ARID1A* Q405*, *CDK4* R24H, *TP53* C277Y

^a^Sequencing has not been performed for the PDC line. The OncoKB Gene Panel is from the matching organoid model.

## Materials and Methods

### Compounds

The drugs and compounds olaparib (NSC753686), talazoparib (NSC767125), berzosertib (NSC777718), elimusertib (NSC800525), nedisertib (NSC802822), VX-984 (NSC792969), AZD-1390 (NSC803789), temozolomide (NSC362856), topotecan (NSC609699), trabectedin (NSC813783), staurosporine (NSC755774), and gemcitabine (NSC613327) were obtained from the NCI Developmental Therapeutics Program Chemical Repository. The FDA-approved anticancer drug set is available from the Developmental Therapeutics Program at https://dtp.cancer.gov/organization/dscb/obtaining/available_plates.htm. The drugs and investigational agents used in this study were demonstrated to be >95% pure by proton nuclear magnetic resonance and LC/MS. The stock solutions were prepared in DMSO (Sigma-Aldrich, catalog no. D2650) at 400-fold the tested concentration and stored at −70°C. Prior to their use, the stock solutions were diluted in fresh medium and the final DMSO concentration in the assay was 0.25% (v/v). All drugs and investigational agents were tested over a range starting from a high concentration at or near the clinical *C*_max_ and decreasing in half-log increments. If the clinical *C*_max_ for an agent had not been determined, the highest concentration tested was 10 μmol/L (Table 1).

### Cell Lines

The patient-derived cancer cell (PDC) lines: 156681-154-R-J1-PDC, 283228-195-R-J1-PDC, 287954-098-R-J1-PDC, 292921-168-R-J2-PDC, 349418-098-R-PDC, 425362-245-T-J1-PDC, 556581-035-R-J1-PDC, and BL0293-F563-PDC were received from the NCI Patient-Derived Models Repository (PDMR, https://pdmr.cancer.gov). The established cell lines HS-SY-2 (RRID:CVCL_8719), MPNST, and SYO-1 (RRID:CVCL_7146) were obtained from the Sloan Kettering Institute. The alveolar soft parts sarcoma cell line ASPS-1 (RRID:CVCL_S738) was established at the NCI and grown from internal seed stocks (33). The following established cell lines were purchased from the ATCC: G-401 (ATCC, catalog no. CRL-1441, RRID:CVCL_0270), SW982 (ATCC, catalog no. HTB-93, RRID:CVCL_1734), VA-ES-BJ (ATCC, catalog no. CRL-2138, RRID:CVCL_1785), NCI-H719 (ATCC, catalog no. CRL-5837, RRID:CVCL_1582), NCI-H196 (ATCC, catalog no. CRL-5823, RRID:CVCL_1509), NCI-H841 (ATCC, catalog no. CRL-5845, RRID:CVCL_1595), NCI-H1618 (ATCC, catalog no. CRL-5879, RRID:CVCL_1480), NCI-H1876 (ATCC, catalog no. CRL-5902, RRID:CVCL_1503), DMS 114 (ATCC, catalog no. CRL-2066; RRID:CVCL_1174), NCI-H211 (ATCC, catalog no. CRL-5824; RRID:CVCL_1529), and SW 1271 (ATCC, catalog no. CRL-2177, RRID:CVCL_1716). The SCLC cell line COR-L88 was purchased from Sigma-Aldrich (catalog no. 92031917-1VL, RRID:CVCL_1141). The NSCLC cell lines NCI-H226 (RRID:CVCL_1544) and NCI-H322M (RRID:CVCL_1557) were established at the NCI and grown from internal seed stocks (34, 35). Pooled donor human umbilical vein endothelial cells (HUVEC; Lonza, catalog no. CC-2519) and human mesenchymal stem cells (hMSC; Lonza, catalog no. PT-2501) were purchased from Lonza.

### Cell Culture

All cells were maintained in an incubator at 37°C and 5% CO_2_ with 95% humidity. The PDC lines were cultured according to standard operating procedures established by the NCI PDMR (https://pdmr.cancer.gov). Briefly all PDCs were thawed and cultured in Matrigel-coated flasks prepared with a working solution of 1X Ham's F-12 nutrient mix, without supplementation (Invitrogen, catalog no. 11765054), 100 U/mL penicillin-streptomycin (Invitrogen, catalog no. 15140122), and 2.5% Matrigel (Corning Inc., catalog no. 354248) for the first three passages, except for 283228-195-R-J1-PDC which must be cultured in uncoated flasks only. All PDCs were cultured in complete DMEM/F-12 media containing advanced DMEM/F-12 (Invitrogen, catalog no. 12634028), 4.9% defined FBS, heat-inactivated (HyClone Laboratories Inc., catalog no. SH30070.03HI), 389 ng/mL hydrocortisone (Sigma-Aldrich, catalog no. H4001), 9.7 ng/mL human EGF recombinant protein (Invitrogen, catalog no. PHG0313), 23.4 μg/mL adenine (Sigma-Aldrich, catalog no. A2786), 97.3 U/mL penicillin-streptomycin (Invitrogen, catalog no. 15140122), 1.9 mmol/L l-glutamine (Invitrogen, catalog no. 25030081), and 9.7 μmol/L Y-27632 dihydrochloride (Tocris Bioscience, catalog no. 1254). The PDCs were cultured in complete DMEM/F12 media without 10 μmol/L Y-27632 dihydrochloride for at least two passages prior to a screen. The alveolar soft parts sarcoma cell line ASPS-1 was cultured in DMEM/Hams F-12 50/50 mix (Corning Inc., catalog no. 10-090-CV) with 10% defined FBS (HyClone Laboratories Inc., catalog no. SH30070.03). The rhabdoid tumor cell line G-401 was cultured in McCoy's 5A (modified) medium (Invitrogen, catalog no. 16600108) with 10% defined FBS (HyClone Laboratories Inc., catalog no. SH30070.03). The sarcoma cell lines: HS-SY-2, SYO-1, and VA-ES-BJ were cultured in DMEM, high glucose (Invitrogen, catalog no. 11965118) with 10% defined FBS (HyClone Laboratories Inc., catalog no. SH30070.03). The SCLC cell lines: NCI-H841, SW 1271, NCI-H1876, NCI-H719, and NCI-H1618 were cultured in DMEM/F-12, HEPES (Invitrogen, catalog no. 11330032), 5% defined FBS (HyClone Laboratories Inc., catalog no. SH30070.03), ITS premix universal culture supplement [insulin (5 μg/mL), transferrin (5 μg/mL), and selenious acid (5 ng/mL)] (Corning Inc., catalog no. 354350), 10 nmol/L hydrocortisone (Sigma-Aldrich, catalog no. H6909), 10 nmol/L β-estradiol (Sigma-Aldrich, catalog no. E2257), and 2 mmol/L l-glutamine (Invitrogen, catalog no. 25030081). The cell lines: NCI-H211, NCI-H196, COR-L88, SW982, MPNST, DMS 114, NCI-H226, and NCI-H322M were cultured in RPMI1640 medium, HEPES (Invitrogen, catalog no. 22400105) with 10% defined FBS (HyClone Laboratories Inc., catalog no. SH30070.03). The pooled donor HUVECs and hMSCs were cultured in endothelial cell growth medium 2 (PromoCell, catalog no. C-22011) and mesenchymal stem cell growth medium 2 (PromoCell, catalog no. C-28009). For all experiments, HUVECs and hMSCs were used at passages ≤5, while the tumor cell lines were used at passages ≤15. Samples of the cell lines were collected at regular intervals throughout the screening process for short tandem repeat profiling and *Mycoplasma* testing by Labcorp (Laboratory Corporation of America Holdings, formerly known as Genetica DNA Laboratories) to confirm their authenticity and integrity.

### High-throughput Drug Combination Screening

Prior to their inoculation into microplates, malignant cells, HUVECs, and hMSCs were removed from T flasks using TrypLE express (Invitrogen, catalog no. 12605036) and harvested by centrifugation for 5 minutes at 233 × *g*. Following removal of the supernatant, the cells were resuspended in fresh medium and counted using a Cellometer auto T4 bright field cell counter (Nexcelom) and trypan blue to distinguish viable cells. Complex tumor spheroids were grown from the mixture of three cell types: 60% malignant cells, 25% HUVECs, and 15% hMSCs as described previously (32). Mixed cell suspensions of 42 μL were dispensed into the wells of 384-well black/clear round-bottom ultra-low attachment (ULA) spheroid microplates (Corning Inc., catalog no. 3830). The suspensions containing malignant cell lines: 283228-195-R-J1-PDC, 287954-098-R-J1-PDC, 349418-098-R-PDC, 425362-245-T-J1-PDC, BL0293-F563-PDC, G-401, HS-SY-2, NCI-H211, NCI-H841, SW982, SYO-1, and VA-ES-BJ comprised 7,450 malignant cells/mL, 1,860 HUVEC/mL, and 1,120 hMSC/mL. Suspensions containing the malignant cell lines: 292921-168-R-J2-PDC, COR-L88, DMS 114, NCI-H1618, and SW 1271 comprised 14,900 malignant cells/mL, 3,700 HUVEC/mL, and 2,240 hMSC/mL. The suspensions containing malignant cell lines: 156681-154-R-J1-PDC, NCI-H226 and NCI-H322M comprised 29,800 malignant cells/mL, 7,450 HUVEC/mL, and 4,480 hMSC/mL. Suspensions containing the malignant cell lines: 556581-035-R-J1-PDC, ASPS-1, NCI-H1876, NCI-H196, and NCI-H719 comprised 59,500 malignant cells/mL, 14,900 HUVEC/mL, and 8,930 hMSC/mL. Finally, MPNST was suspended at 119,000 cells/mL with 29,800 HUVEC/mL and 17,900 hMSC/mL. Following inoculation, the microplates were transferred to a Cytomat 10 incubator (Thermo Fisher Scientific) and maintained at 37°C and 5% CO_2_ with 95% humidity. Three days after inoculation, test agents or controls were delivered to the wells of microplates. The approved and investigational anticancer agents, prepared as 400 × DMSO stock solutions, were first diluted 50-fold in media, and 6 μL were subsequently transferred to the appropriate wells of microplates containing 42 μL cell suspension using a Tecan Freedom EVO 200 base unit (Tecan) to achieve a 1x final concentration. All anticancer agents and their combinations were tested in quadruplicate. In addition, each microplate included a DMSO vehicle control (*n* = 16) and a cytotoxicity control (1 μmol/L staurosporine and 3 μmol/L gemcitabine, *n* = 20). After delivery of the test agents and controls, the microplates were returned to the incubator for 7 days. Ten days after inoculation, the assay was completed with the addition of 20 μL CellTiter-Glo three-dimensional (3D; Promega, catalog no. G9683) to each well. Representative brightfield images of DMSO-treated wells on day 10 are shown in Supplementary Fig. S1. Next, the microplates were placed on a microplate shaker for 5 minutes. After 25 minutes of incubation at room temperature, luminescence was measured as a surrogate indicator of cell viability using a PHERAstar FSX microplate reader (BMG LABTECH) or an EnSpire multimode plate reader (PerkinElmer).

### Data Analysis

Luminescence measurements from the screen were exported as comma separated values (CSV) files and imported into custom Excel spreadsheets (Microsoft) for analysis. The raw luminescence data were evaluated for quality control, filtered for outliers, and converted to percent viability by normalizing to the DMSO (vehicle-treated) control. Concentration–response data were fit to the four-parameter logistic equation using the Solver Add-In in Excel. The Bliss independence model states that if two drugs have independent activities, then the viability for the combination is equal to the product of the viability of the two single agents (36). Synergy among two compounds is indicated by a lower observed percent viability than predicted by Bliss independence, whereas antagonism is indicated by a greater observed percent viability than predicted. All data were analyzed and graphed using GraphPad Prism version 9.4.1 for Windows (GraphPad Software).

### Data Availability

All data are accessible via the PubChem BioAssay public database (AID 1845193; AID 1845194; AID 1845195; AID 1845196; AID 1845197; AID 1845198; AID 1845199; AID 1845200; AID 1845201; AID 1845202; AID 1845203; AID 1845204; AID 1845205; AID 1845206; AID 1845207; AID 1845208; AID 1845209; AID 1845210; AID 1845211; AID 1845212; AID 1845213; AID 1845215; AID 1845216; AID 1845217; AID 1845218; AID 1845219, also see Supplementary Table S2).

## Results

**FIGURE 1:**
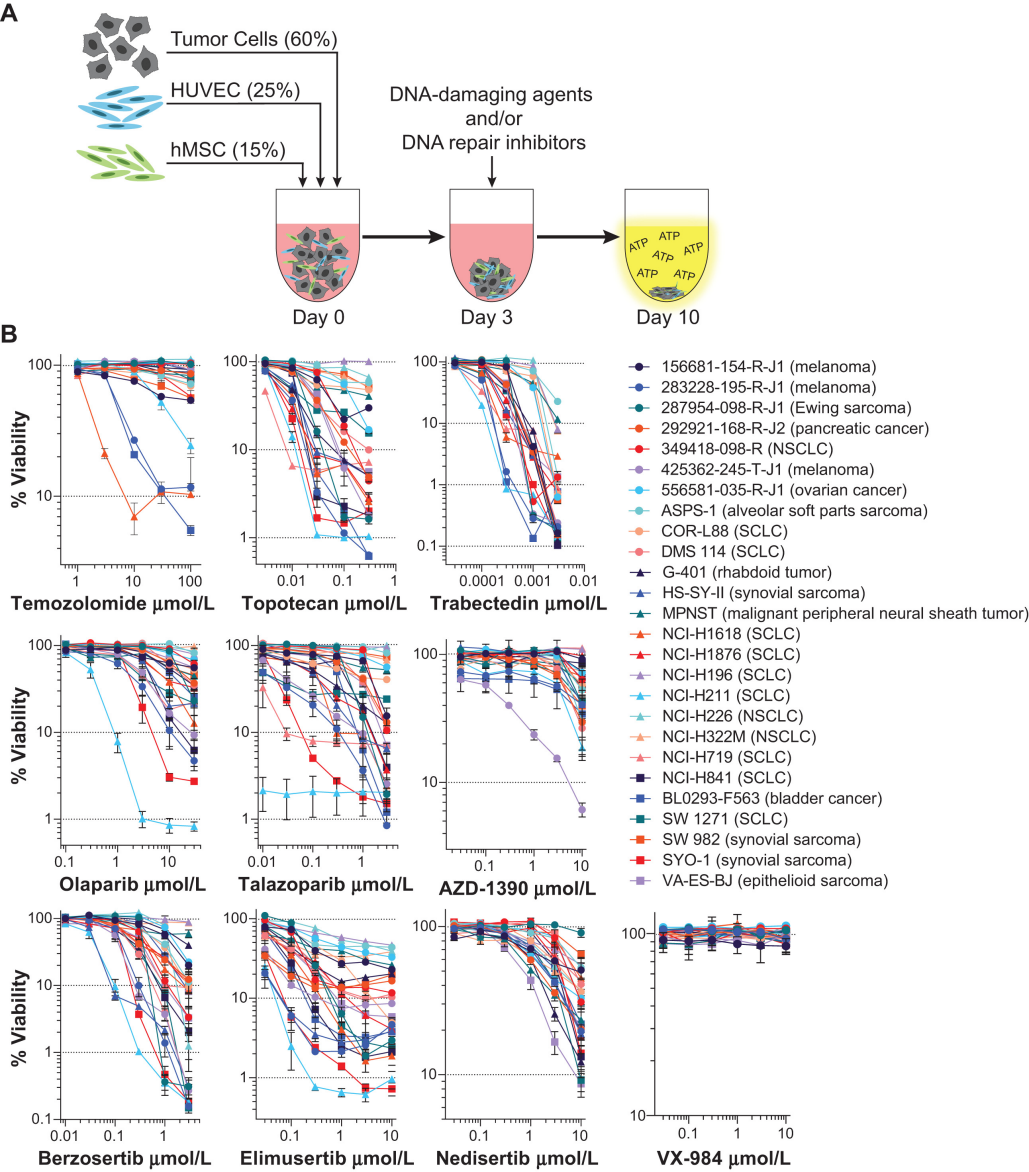
Multicellular tumor spheroid screening assay. **A,** A schematic of the complex tumor spheroid screening assay. Patient-derived or established malignant cell lines, HUVECs, and hMSCs are dispensed into 384-well, round bottom, ULA plates. After 3 days of spheroid growth, single agents or drug combinations are added. The concentration of ATP is measured as luminescence with CellTiter-Glo 3D 7 days later as a surrogate for cell viability. **B,** Concentration–response curves are shown for all complex tumor spheroid models (*n* = 26) treated with the three DNA-damaging drugs and seven DNA repair inhibitors (mean ± SD, *n* = 4 technical replicates).

Three-dimensional multicellular tumor spheroids have become increasingly adopted, because they more accurately mimic the structure of solid tumors and the tumor microenvironment but are also suitable for automated high-throughput screening (37). Here, the complex tumor spheroid model shown in Fig. 1A was utilized, wherein spheroids were grown from human malignant cells, HUVECs, and hMSCs. Both patient-derived and established tumor cell lines were incorporated into the complex tumor spheroids (Table 2). Following spheroid growth over 3 days, DNA-damaging drugs and DNA repair inhibitors were added alone or in combination, and cell viability was subsequently measured by CellTiter-Glo 3D after 7 days of exposure. The list of DNA-damaging drugs and the DNA repair inhibitors tested is shown in Table 1, including their current clinical status, molecular target, and clinical *C*_max_, if known.

Prior to evaluating combinations, the single-agent activity of each compound was investigated. Figure 1B shows the concentration–response curves of the three DNA-damaging drugs and seven DNA repair inhibitors for the 26 human malignant cell lines grown as multicellular spheroids. The DNA-damaging drugs showed marked differential cytotoxicity and potency among the spheroids. At concentrations up to the clinical *C*_max_ (37.6 μmol/L) of temozolomide, only three of 26 complex tumor spheroids reach approximately 90% or one log of cytotoxicity. Topotecan exhibited broad antiproliferative activity across the panel of complex tumor spheroids with IC_50_ values spanning more than two logs from 3 to >300 nmol/L. Trabectedin is one of the most potent approved anticancer drugs. The IC_50_ values for trabectedin ranged from 0.1 nmol/L to near its clinical *C*_max_ of 2.4 nmol/L with approximately three logs of cytotoxicity in the most sensitive complex tumor spheroid models. Among the DNA repair inhibitors, the ATR inhibitors, berzosertib and elimusertib, showed the greatest activity with approximately two logs of cytotoxicity at the highest concentrations tested. Talazoparib is a more potent PARP inhibitor than olaparib, yet both PARP inhibitors demonstrated some selectivity for the complex tumor spheroids grown from the SCLC line NCI-H211 and the synovial sarcoma line SYO-1. Aside from the complex tumor spheroids grown from the patient-derived melanoma line 425362-245-T-J1, the ATM inhibitor AZD-1390 only showed activities near 10 μmol/L. For the DNA-PK inhibitors, nedisertib showed little activity below 1 μmol/L and VX-984 was noncytotoxic throughout the concentration range.

### PARP Inhibitors in Combination with DNA-damaging Agents

**FIGURE 2:**
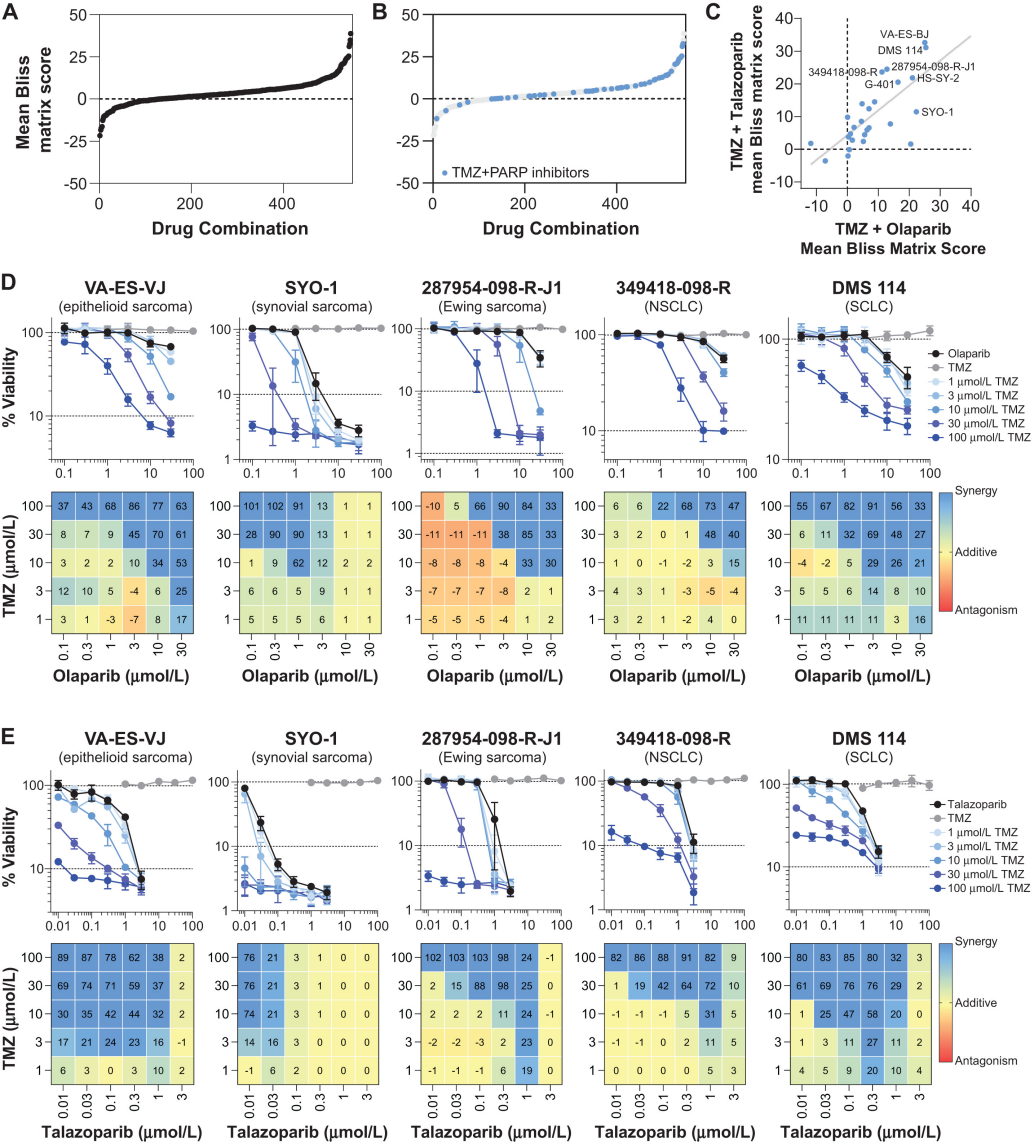
Combination of PARP inhibitors and temozolomide. **A,** Mean Bliss matrix scores were calculated from each combination's concentration matrix [(5 concentrations of drug A) × (6 concentrations of drug B) = (30 combination concentrations)]. The scores (*n* = 546) are graphed for all combinations of DNA-damaging drugs with DNA repair inhibitors tested (*n* = 21) in all complex tumor spheroid models (*n* = 26). **B,** The same data shown in A) but combinations of the DNA-damaging drug temozolomide with either PARP inhibitor olaparib or talazoparib are highlighted in blue (*n* = 52), whereas all other combinations are shown in light gray. **C,** A scatter plot of the mean Bliss matrix scores from the temozolomide combinations with olaparib and talazoparib (Pearson *r* = 0.73, two-tailed *P* < 0.0001). Concentration–response curves (top, mean ± SD, *n* = 4 technical replicates) from combinations of temozolomide with either olaparib (**D**) or talazoparib (**E**) and corresponding mean Bliss score plots (bottom, *n* = 4 technical replicates) showing the scores from each combination's concentration matrix and colored as a heat map (blue indicates synergy; yellow indicates additivity; red indicates antagonism). Data are shown for complex tumor spheroids grown with the malignant cell lines (from left): VA-ES-VJ (epithelioid sarcoma), SYO-1 (synovial sarcoma), 287954-098-R-J1 (Ewing sarcoma), 349418-098-R (NSCLC), and DMS 114 (SCLC).

Next, the activities of DNA repair inhibitors in combination with DNA-damaging drugs were assessed. Combination activities were evaluated by the Bliss independence model (36), which compares the combination response observed with the combination response predicted from the observed activities of the individual agents. Bliss scores near zero indicate an additive effect, wherein the observed response resembles the predicted. Highly positive Bliss scores indicate synergy, while a negative score indicates antagonism. Mean Bliss matrix scores were calculated from each combination's concentration matrix [(5 concentrations of drug A) × (6 concentrations of drug B) = (30 combination concentrations)]. Figure 2A provides an overview of mean Bliss matrix scores (*n* = 546) from all drug combinations tested (*n* = 21) across all complex tumor spheroid models (*n* = 26). One of the top-ranking combinations from the mean Bliss matrix scores was temozolomide combined with either of the two PARP inhibitors (Supplementary Table S1). As shown in Fig. 2B, most of the temozolomide combinations with the PARP inhibitors resulted in highly positive mean Bliss matrix scores (blue circles) suggesting greater than additive or synergistic responses. The mean Bliss scores for each DNA-damaging drug in combination with olaparib or talazoparib were represented as heat maps across the entire concentration matrix for all 26 multicellular complex tumor spheroids (Supplementary Fig. S2). While either topotecan or trabectedin in combination with the PARP inhibitors showed some synergistic activity, the drug interactions were mainly additive. There was a linear correlation (Pearson *r* = 0.73, two-tailed *P* < 0.0001) between the mean Bliss matrix scores calculated from combinations of temozolomide with olaparib and talazoparib across all complex tumor spheroid models (Fig. 2C). Concentration–response curves and corresponding mean Bliss score plots for the combination of temozolomide with both PARP inhibitors are shown for five complex tumor spheroid models (Fig. 2D and E). Compared with the PARP inhibitors, temozolomide showed no single-agent activity in the selected complex tumor spheroid models under the assay conditions, yet the combinations resulted in synergistic cytotoxicity (Fig. 2D and E). In general, the synergy was observed at higher concentrations of temozolomide, and similar results were observed amongst several different tumor types.

### ATM Inhibitor in Combination with DNA-damaging Agents

**FIGURE 3:**
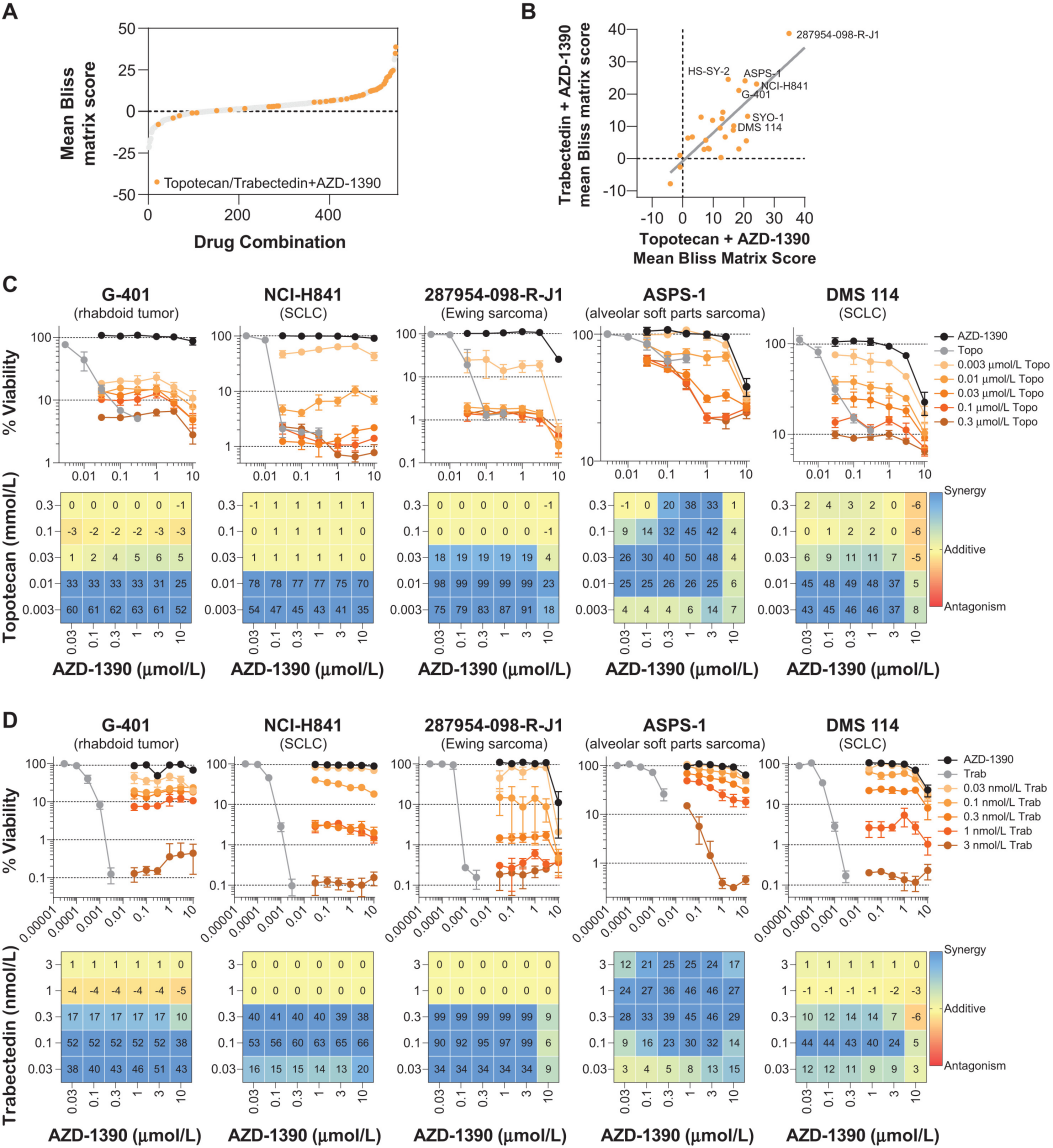
Combination of AZD-1390 and topotecan or trabectedin. **A,** Mean Bliss matrix scores (*n* = 546) were calculated from the concentration matrix [(5 concentrations of drug A) × (6 concentrations of drug B) = (30 combination concentrations)] of each combination tested (*n* = 21) in all complex tumor spheroid models (*n* = 26). The mean Bliss matrix scores are highlighted from combinations of the ATM inhibitor AZD-1390 with DNA-damaging drugs topotecan or trabectedin (*n* = 52, orange), whereas the scores from all other combinations are shown in light gray. **B,** A scatter plot of the mean Bliss matrix scores from the AZD-1390 combinations with topotecan and trabectedin (Pearson *r* = 0.77, two-tailed *P* < 0.0001). Concentration–response curves (top, mean ± SD, *n* = 4 technical replicates) from combinations of AZD-1390 with either topotecan (**C**) or trabectedin (**D**) and corresponding mean Bliss score plots (bottom, *n* = 4 technical replicates) showing the scores from each combination's concentration matrix and colored as a heat map (blue indicates synergy; yellow indicates additivity; red indicates antagonism). Data are shown for complex tumor spheroids grown with the malignant cell lines (from left): G-401 (rhabdoid tumor), NCI-H841 (SCLC), 287954-098-R-J1 (Ewing sarcoma), ASPS-1 (alveolar soft part sarcoma), and DMS 114 (SCLC).

Additional top-ranking combinations based on the mean Bliss matrix score were topotecan or trabectedin in combination with the ATM inhibitor, AZD-1390 (Fig. 3A; Supplementary Table S1). An analysis of the mean Bliss scores across each combination's concentration matrix (Supplementary Fig. S3) clearly indicated that synergy most frequently occurred with the combination of AZD-1390 and either topotecan or trabectedin. There were no apparent cytotoxicity differences in either the combination of AZD-1390 with temozolomide or AZD-1390 alone. In addition, although topotecan and trabectedin damage DNA in different ways, a correlation (Pearson *r* = 0.77, two-tailed *P* < 0.0001) was observed between the mean Bliss matrix scores for topotecan or trabectedin in combination with AZD-1390 across all complex tumor spheroid models (Fig. 3B). The synergistic activities from the combination of AZD-1390 with topotecan were most evident at lower concentrations of topotecan as shown for five complex tumor spheroid models (Fig. 3C). Similarly, lower concentrations of trabectedin also demonstrated synergistic activities with AZD-1390 (Fig. 3D). The depth of cytotoxicity was greater when AZD-1390 was combined with trabectedin than with topotecan. Although synergy was observed, ASPS-1 showed the least depth of cytotoxicity among the five selected complex tumor spheroid models (Fig. 3D).

### ATR Inhibitors in Combination with DNA-damaging Agents

**FIGURE 4:**
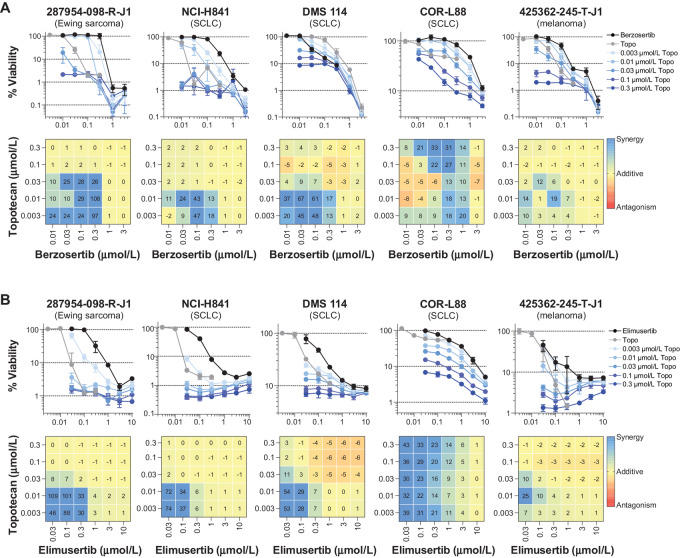

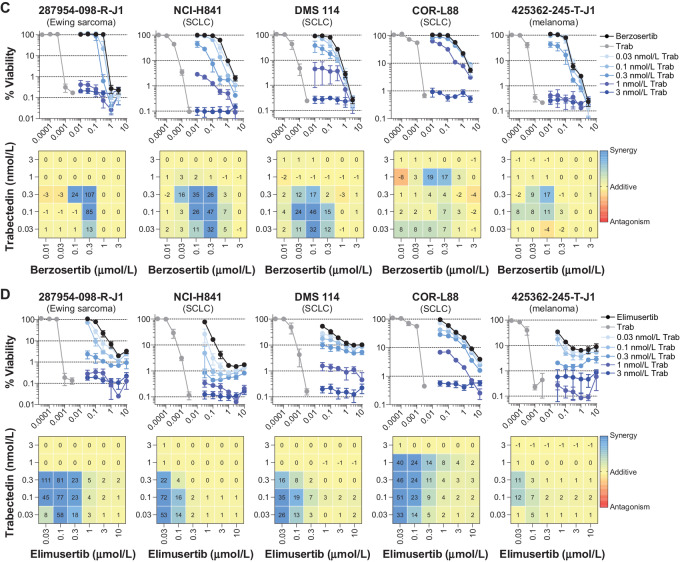
Combination of ATR inhibitors with topotecan or trabectedin. Concentration–response curves (top, mean ± SD, *n* = 4 technical replicates) with corresponding mean Bliss score plots (bottom, *n* = 4 technical replicates) showing the scores from each combination's concentration matrix and colored as a heat map (blue indicates synergy; yellow indicates additivity; red indicates antagonism). Combinations of the DNA-damaging drug topotecan with the ATR inhibitors berzosertib (**A**) and elimusertib (**B**). Combinations of the DNA-damaging drug trabectedin with either berzosertib (**C**) or elimusertib (**D**). Data are shown for complex tumor spheroids grown with the malignant cell lines (from left): 287954-098-R-J1 (Ewing sarcoma), NCI-H841 (SCLC), DMS 114 (SCLC), COR L88 (SCLC), and 425362-245-T-J1 (melanoma).

Combinations of the three DNA-damaging drugs with the ATR inhibitors, berzosertib or elimusertib, predominantly resulted in additive or slightly synergistic cytotoxicity across each combination's concentration matrix (Supplementary Fig. S4). Like the ATM inhibitor, combinations of the ATR inhibitors with temozolomide did not show a greater response than the single agents in nearly all complex tumor spheroid models. Some greater than additive responses were observed in several complex tumor spheroid models for the combination of topotecan or trabectedin with either ATR inhibitor. Figure 4A and B highlight five of the complex tumor spheroid models that demonstrated notable combination activities with topotecan and the ATR inhibitors. Complex tumor spheroid models grown from the patient-derived Ewing sarcoma line (287954-098-R-J1) and three SCLC lines (NCI-H841, DMS 114, and COR-L88) showed synergy between topotecan and both ATR inhibitors. However, the same combination was mainly additive in complex tumor spheroid models grown from the patient-derived melanoma line 425362-245-T-J1. Similar results were observed with trabectedin and the ATR inhibitors (Fig. 4C and D), although the depth of cytotoxicity was greater.

### DNA-PK Inhibitors in Combinations with DNA-damaging Agents

**FIGURE 5:**
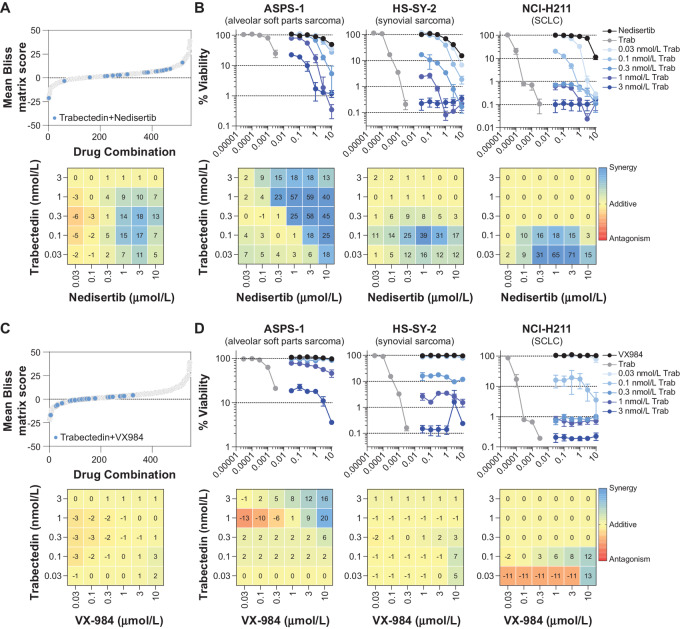
Combination of DNA-PK inhibitors with trabectedin. **A,** Top, Mean Bliss matrix scores (*n* = 546) were calculated from the concentration matrix [(5 concentrations of drug A) × (6 concentrations of drug B) = (30 combination concentrations)] of each combination tested (*n* = 21) in all complex tumor spheroid models (*n* = 26). Mean Bliss matrix scores from the combination of the DNA-damaging drug trabectedin with the DNA-PK inhibitor nedisertib are highlighted in blue, whereas all other combinations are shown in light gray. **A,** Bottom, A mean Bliss score plot (*n* = 104) calculated from the combination concentration matrix of trabectedin with nedisertib across all complex tumor spheroid models (*n* = 26). The data are colored as a heat map, where blue indicates synergy, yellow indicates additivity, and red indicates antagonism. **B,** Concentration–response curves (top, mean ± SD, *n* = 4 technical replicates) from combinations of trabectedin with nedisertib and corresponding mean Bliss score plots (bottom, *n* = 4 technical replicates) showing the scores from each combination's concentration matrix and colored as a heat map. Data are shown for complex tumor spheroids grown with the malignant cell lines (from left): ASPS-1 (alveolar soft part sarcoma), HS-SY-2 (synovial sarcoma), and NCI-H211 (SCLC). **C,** Top, Mean Bliss matrix scores are highlighted from the combination of trabectedin with the DNA-PK inhibitor VX-984 across all complex tumor spheroid models (blue, *n* = 26), whereas all other combinations are shown in light gray. **C,** Bottom, A mean Bliss score plot (*n* = 104) calculated from the combination concentration matrix of trabectedin with VX-984 across all complex tumor spheroid models (*n* = 26). **D,** Concentration–response curves (top, mean ± SD, *n* = 4 technical replicates) from combinations of trabectedin with VX-984 and corresponding mean Bliss score plots (bottom, *n* = 4 technical replicates) showing the scores from each combination's concentration matrix and colored as a heat map. Data are shown for complex tumor spheroids grown with the malignant cell lines (from left): ASPS-1 (alveolar soft part sarcoma), HS-SY-2 (synovial sarcoma), and NCI-H211 (SCLC).

Finally, combinations of the three DNA-damaging drugs with the DNA-PK inhibitors, nedisertib and VX-984, were examined (Supplementary Fig. S5). Temozolomide did not alter the cytotoxicity of the DNA-PK inhibitors. Topotecan primarily demonstrated additive responses with nedisertib and VX-984, which was inactive as a single agent. An analysis of trabectedin in combination with nedisertib revealed primarily additive responses and some degree of synergy based on the mean Bliss scores (Fig. 5A). Concentration–response curves and mean Bliss score plots are shown in Fig. 5B for three selected complex tumor spheroid models treated with the combination of trabectedin and nedisertib. These results suggest that DNA-PK might be involved in repair of the DNA damage caused by trabectedin, yet similar results were not observed with the other DNA-PK inhibitor, VX-984 (Fig. 5C and D), which could be due to the inactivity of VX-984 in the assay.

## Discussion

While interpreting the data, it is important to consider some of the limitations of this study. All complex tumor spheroids were grown from a fixed ratio of 60% malignant cells, 25% HUVECs, and 15% hMSCs according to a standardized protocol. Spheroids grown by this method from the patient-derived pancreatic adenocarcinoma cell line K24384-001-R were characterized extensively (32). However, complex tumor spheroid morphologies varied among malignant cell lines (Supplementary Fig. S1) and there are likely differences in the growth rates of component cells that affect the overall composition and responsiveness to anticancer agents (32). Among the many types and subtypes of solid cancer, stromal compartments and their compositions within the tumor microenvironment vary which can impact the therapeutic response (38). Within the framework of these constraints, this study examines responses from a wide range of patient-derived or established malignant cell lines grown as complex, multicellular tumor spheroids following treatment with combinations of DNA repair inhibitors and DNA-damaging drugs. The results of this study are consistent with published data from other preclinical assays as discussed in detail below.

The clinical efficacy of traditional DNA-damaging anticancer drugs can be significantly influenced by the cellular capacity of the DNA damage response. For example, upregulation of DNA damage response mechanisms or relying on alternative pathways provides tumor cells with a means to recover from damage and avoid cell death. Therefore, it has been rationalized that pharmacologic inhibition of the relevant DNA repair pathways may improve the efficacy of DNA damage–based cancer therapies. Here, we sought to identify DNA repair inhibitors that can potentiate the cytotoxicity of several DNA-damaging drugs. The major observation from this study was that DNA-damaging drugs in combination with DNA repair inhibitors primarily showed additive activities, with several combinations showing notable synergistic activities.

Synergistic effects were evident in our results with the PARP inhibitors, olaparib and talazoparib, in simultaneous combination with temozolomide (Fig. 2D and E). This is in line with previous preclinical studies in 10 glioblastoma multiforme cancer stem cell lines that demonstrated synergy between temozolomide and PARP inhibitor (39). While cytotoxic temozolomide-induced O6MeG lesions are reversed by MGMT, the more abundant N7MeG (N7-methylguanine) and N3MeA (N3-methyladenine) adducts are repaired by the BER pathway in a process that requires PARP (40). Thus, the inhibition of PARP-mediated BER would be expected to give rise to unrepaired and potentially lethal temozolomide-induced N7MeG and N3MeA lesions leading to enhanced temozolomide cytotoxicity. More recently, it was demonstrated that PARylation of MGMT by PARP is critical for repairing O6MeG adducts, suggesting the involvement of PARP in both BER and MGMT-mediated DNA repair of temozolomide-induced DNA damage (41). Given the success in preclinical cancer models (42–44), the temozolomide/PARP inhibitor combination has been and continues to be evaluated in clinical trials. Most clinical studies were initially focused on glioblastoma, given temozolomide is the first-line therapy; however, ongoing clinical studies for the temozolomide/PARP inhibitor regimen have expanded to additional tumor types, including SCLC (NCT04434482), renal cell cancer (NCT04603365), Ewing sarcoma, or rhabdomyosarcoma (NCT01858168), as well as advanced stage rare cancers (NCT05142241).

ATM inhibition has been shown to sensitize cells to ionizing radiation and to DNA DSB-inducing drugs including topotecan (13). Among the complex tumor spheroids evaluated in this study, synergistic activities were frequently observed when the ATM inhibitor AZD-1390 was combined with topotecan or trabectedin (Fig. 3). A strong correlation between the mean Bliss matrix scores of these combinations was observed across all the complex tumor spheroid models (Fig. 3B, Pearson *r* = 0.77, two-tailed *P* < 0.0001), despite the different DNA-damaging mechanisms of topotecan and trabectedin. These results are consistent with other published studies. The recently described ATM-selective inhibitor M4076 showed synergistic activities with topotecan or irinotecan in a panel of 34 cancer cell lines. In addition, the combination of irinotecan and M4076 showed substantial activity in the SW620 xenograft model compared with either single agent (45). AZD-1390, which is an oral agent and crosses the blood–brain barrier, is currently in phase I clinical trial with radiation therapy for various types of brain tumors (NCT03423628) and in NSCLC (NCT04550104).

A number of preclinical studies support the combination of ATR inhibitors with DNA-damaging agents. The sensitization of several ovarian cancer cell lines to topotecan was demonstrated either by inhibition of ATR with VE-821 or depletion of ATR by RNAi (46). Subsequently, an siRNA screen targeting approximately 7,000 human genes in the breast cell line MDA-MB-231 identified *ATR* as a top candidate for camptothecin synthetic lethality (47). Further studies revealed antiproliferative synergy between VE-821 and camptothecin in MDA-MB-231, HT-29, and HCT-116 cell lines. Synergy was also observed between berzosertib and SN-38 (the active metabolite of irinotecan) in the COLO205 colorectal cancer cell line. Finally, the potentiation of irinotecan by berzosertib was demonstrated in mice bearing subcutaneous COLO205 tumors (47). As with berzosertib, the recently reported ATR inhibitor M4344 demonstrated synergistic cytotoxicity in combination with topotecan or irinotecan in established cell lines, patient-derived prostate tumor organoids, and SCLC tumor xenograft models (48). Preclinical studies of elimusertib combined with DNA-damaging agents, including SN-38, showed additive to synergistic activity in cancer cell lines of different genetic backgrounds and tumor types. Notably, the combination of elimusertib with SN-38 showed strong synergistic activity in the established cell lines: HT-29, LOVO, PC-3, HT-144, MDA-MB-231 (49). In the current study, both ATR inhibitors berzosertib and elimusertib potentiated the cytotoxicity of the DNA-damaging drugs topotecan and trabectedin (Fig. 4). Both additive and synergistic activities were observed for these combinations in complex tumor spheroid models grown with the SCLC cell lines: NCI-H841, DMS 114, and COR L88, as well as the patient-derived melanoma cell line 425362-245-T-J1 and the Ewing sarcoma cell line 287954-098-R-J1. The combination of berzosertib and topotecan is currently in phase I/II trials (NCT02487095, NCT03896503, NCT04768296) for patients with SCLC (50, 51). Berzosertib is also in phase I/II clinical trials with lurbinectedin, a synthetic derivative of the natural product trabectedin, (NCT04802174) and sacituzumab govitecan (NCT04826341) in patients with SCLC. A phase I trial with elimusertib demonstrated tolerability as well as antitumor activity in heavily pretreated patients with various advanced solid tumors, particularly those with deleterious variants to ATM and/or the loss of ATM protein (52). Elimusertib is currently in several phase I clinical trials including with gemcitabine in ovarian and pancreatic cancer (NCT04616534) and with FOLFIRI in stomach and intestinal cancer (NCT04535401).

DNA-PK is a multifunctional serine-threonine protein kinase and a component of multiprotein complexes that facilitate NHEJ and transcriptional programs (53, 54). High DNA-PK expression and activity is linked to poor outcomes in a number of tumor types (55, 56). Several preclinical studies have evaluated combinations of DNA-PK inhibitors and DNA-damaging agents or radiation. The combination of nedisertib with pegylated liposomal doxorubicin showed enhanced activity compared with the single agents in ovarian cancer xenografts (57). VX-984 enhanced the radiosensitivity of glioblastoma cells grown *in vitro* and as orthotopic xenografts (58). Boucher and colleagues reported potent *in vitro* cytotoxicity and strong synergy for combinations of VX-984 with either doxorubicin or etoposide in established cancer cell lines and primary tumor explants (59). In the current study, combinations of either nedisertib or VX-984 with the DNA-damaging drugs temozolomide, topotecan, and trabectedin primarily showed additive cytotoxicity among the complex tumor spheroids (Supplementary Fig. S5A and S5B). However, some synergistic activities were observed for the combination of nedisertib and trabectedin in complex tumor spheroids grown with the alveolar soft part sarcoma cell line ASPS-1, the synovial sarcoma cell line HS-SY-2, and the SCLC cell line NCI-H211 (Fig. 5A and B). Nedisertib is currently in phase I trials as a single agent (NCT02316197) and in combination with radiotherapy and cisplatin in advanced solid tumors (NCT02516813). In addition, the combination of nedisertib and radiotherapy is being evaluated in phase I trials with MGMT unmethylated glioblastoma or gliosarcoma (NCT04555577), advanced head and neck cancer (NCT04533750) and phase I/II trials in patients with pancreatic cancer (NCT04172532). The combination of nedisertib and doxorubicin is in phase I trials in patients with recurrent with high- or low-grade ovarian cancer (NCT04092270). Nedisertib is also in phase I/II trials with capecitabine and radiotherapy in patients with locally advanced rectal cancer (NCT03770689). VX-984 is currently being tested in a first in human phase I study with doxorubicin (NCT02644278).

It has long been recognized that cells, by the necessity to survive, efficiently and rapidly repair the DNA damage produced by anticancer drugs. In early attempts, the potential promise of DNA repair inhibitors was thwarted by equal activity occurring in normal cells and malignant cells; thus, no therapeutic benefit was achieved. The success of the PARP inhibitors in tumors with *BRCA1/2* variants stimulated interest in designing and testing specific DNA repair inhibitors. These agents have demonstrated activity in preclinical cell-based models and in tumor-bearing mice and are now in early clinical trials. The investigational DNA repair inhibitors discussed herein as well as others are highly selectively targeted and the genomics of malignant tumors are better understood than in the past, therefore, clinical success of DNA-damaging drugs in combination with DNA repair inhibitors are plausible.

## Supplementary Material

Supplementary Figure 1 Figure S1. Representative brightfield images for assay optimized cell densities from a DMSO-treated well on Day 10.

Supplementary Figure 2 Figure S2. Heat maps of Bliss synergy scores across the combination dose-response matrices for all twenty-six cell lines grown as multicellular complex spheroids exposed to each DNA damaging agent (A, TMZ; B, topotecan; C, trabectedin) in combination with PARP inhibitors, olaparib or talazoparib.

Supplementary Figure 3 Figure S3. Heat maps of Bliss synergy scores across the combination dose-response matrices for all twenty-six cell lines grown as multicellular complex spheroids exposed to each DNA damaging agent (A, TMZ; B, topotecan; C, trabectedin) in combination with the ATM inhibitor, AZD1390.

Supplementary Figure 4 Figure S4. Heat maps of Bliss synergy scores across the combination dose-response matrices for all twenty-six cell lines grown as multicellular complex spheroids exposed to each DNA damaging agent (A, TMZ; B, topotecan; C, trabectedin) in combination with ATR inhibitors, berzosertib or elimusertib.

Supplementary Figure 5 Figure S5. Heat maps of Bliss synergy scores across the combination dose-response matrices for all twenty-six cell lines grown as multicellular complex spheroids exposed to each DNA damaging agent (A, TMZ; B, topotecan; C, trabectedin) in combination with DNA-PK inhibitors, nedisertib or VX-984.

Supplementary Table 1 Table S1. Top-ranking drug combinations by mean Bliss matrix score.

Supplementary Table 2 Table S2. Links to the PubChem assays.
