# Supplementary material for: Multicellular Complex Tumor Spheroid Response to DNA Repair Inhibitors in Combination with DNA-damaging Drugs
Source: Cancer Res Commun. 2023 Aug 25;3(8):1648–61. doi: 10.1158/2767-9764.CRC-23-0193 (PMC10452929; doi:10.1158/2767-9764.CRC-23-0193)
Supplement: Supplementary Figure 1 — Figure S1. Representative brightfield images for assay optimized cell densities from a DMSO-treated well on Day 10. [file crc-23-0193-s01.pdf]

Supplementary Figure S1

Model	# Tumor Cells/Well	# HUVEC Cells/Well	# hMSC/Well	Brightfield Image
156681-154-R-J1-PDC	1250	313	188	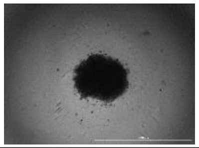
283228-195-R-J1-PDC	313	78	47	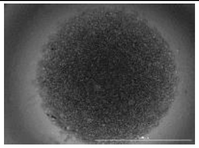
287954-098-R-J1-PDC	313	78	47	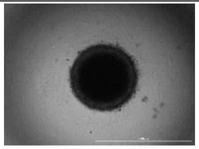
292921-168-R-J2-PDC	625	156	94	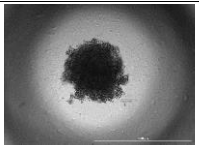
349418-098-R-PDC	313	78	47	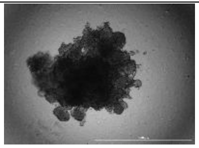
425362-245-T-J1-PDC	313	78	47	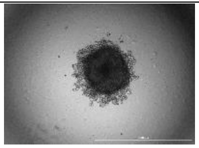

Supplementary Figure S1 continued

Model	# Tumor Cells/Well	# HUVEC Cells/Well	# hMSC/Well	Brightfield Image
556581-035-R-J1-PDC	2500	625	375	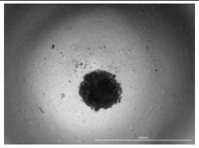
BL0293-F563-PDC	313	78	47	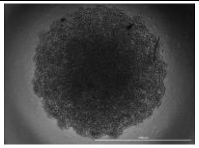
COR L88	625	156	94	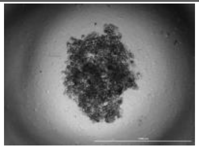
DMS-114	625	156	94	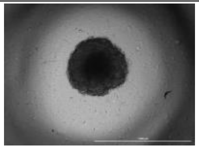
ASPS-1	2500	625	375	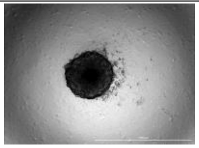
NCI-H196	2500	625	375	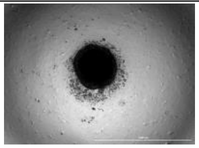

Supplementary Figure S1 continued

Model	# Tumor Cells/Well	# HUVEC Cells/Well	# hMSC/Well	Brightfield Image
NCI-H211	313	78	47	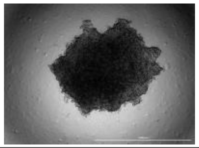
NCI-H719	2500	625	375	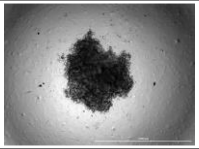
NCI-H1618	625	156	94	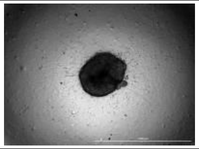
NCI-H1876	2500	625	375	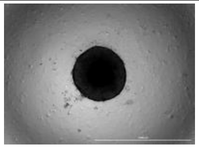
MPNST	5000	1250	750	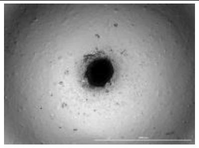
SW 1271	625	156	94	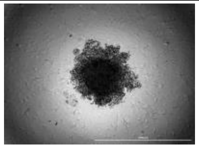

Figure S1. Representative brightfield images for assay optimized cell densities from a DMSO-treated well on Day 10.
