# Supplementary material for: Multicellular Complex Tumor Spheroid Response to DNA Repair Inhibitors in Combination with DNA-damaging Drugs
Source: Cancer Res Commun. 2023 Aug 25;3(8):1648–61. doi: 10.1158/2767-9764.CRC-23-0193 (PMC10452929; doi:10.1158/2767-9764.CRC-23-0193)
Supplement: Supplementary Figure 5 — Figure S5. Heat maps of Bliss synergy scores across the combination dose-response matrices for all twenty-six cell lines grown as multicellular complex spheroids exposed to each DNA damaging agent (A, TMZ; B, topotecan; C, trabectedin) in combination with DNA-PK inhibitors, nedisertib or VX-984. [file crc-23-0193-s05.pdf]

Supplementary Figure S5

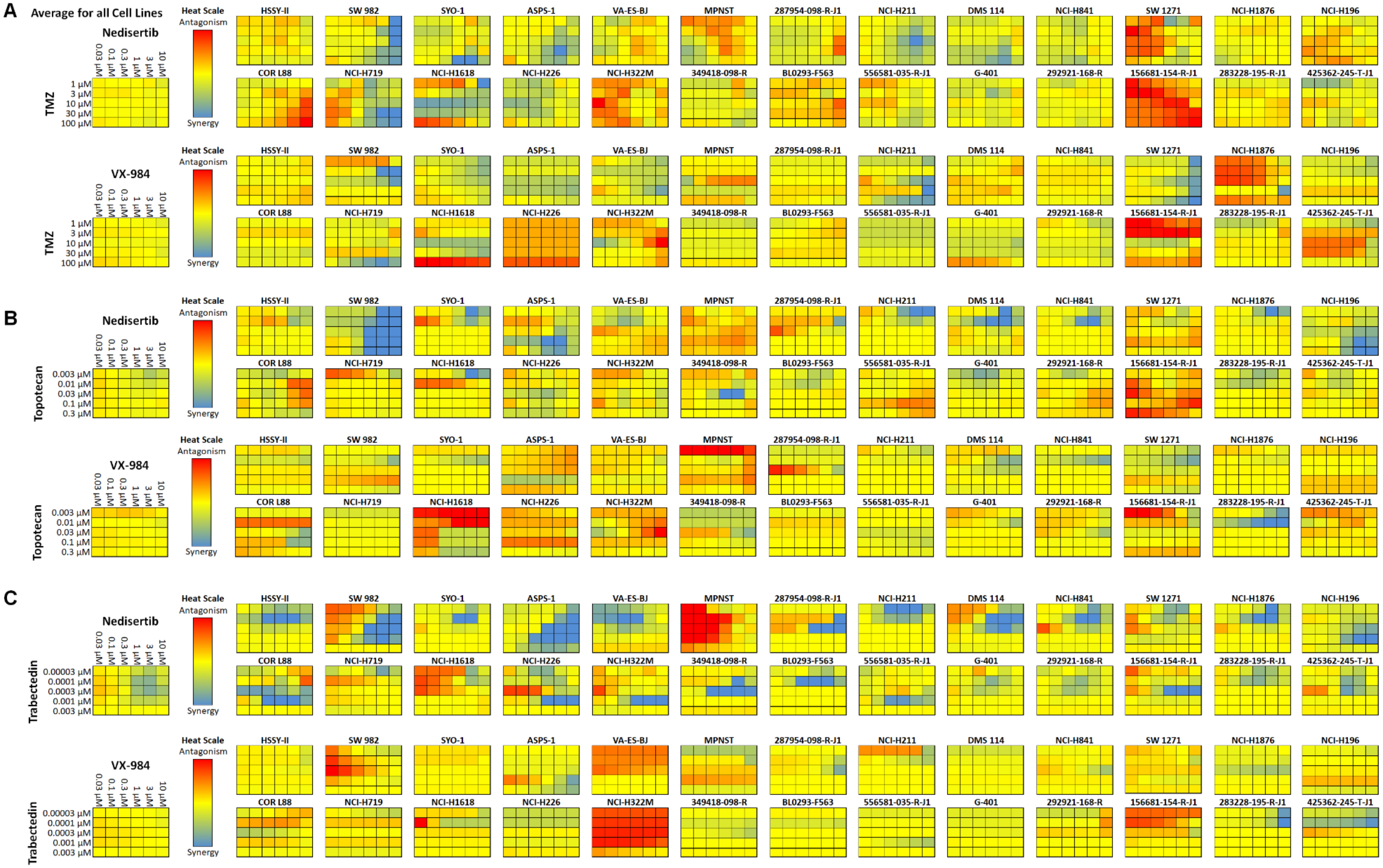

Figure S5. Heat maps of Bliss synergy scores across the combination dose-response matrices for all twenty-six cell lines grown as multicellular complex spheroids exposed to each DNA damaging agent (A, TMZ; B, topotecan; C, trabectedin) in combination with DNA-PK inhibitors, nedisertib or VX-984.
