# Supplementary material for: Multicellular Complex Tumor Spheroid Response to DNA Repair Inhibitors in Combination with DNA-damaging Drugs
Source: Cancer Res Commun. 2023 Aug 25;3(8):1648–61. doi: 10.1158/2767-9764.CRC-23-0193 (PMC10452929; doi:10.1158/2767-9764.CRC-23-0193)
Supplement: Supplementary Table 1 — Table S1. Top-ranking drug combinations by mean Bliss matrix score. [file crc-23-0193-s06.pdf]

Supplementary Table S1. Top-ranking drug combinations by mean Bliss matrix score

Cell line	DNA repair inhibitor	DNA damaging agent	Mean Bliss matrix score
287954-098-R-J1	AZD-1390	trabectedin	38.8
287954-098-R-J1	AZD-1390	topotecan	34.9
VA-ES-BJ	talazoparib	temozolomide	32.7
DMS 114	talazoparib	temozolomide	31.2
DMS 114	olaparib	temozolomide	25.4
VA-ES-BJ	olaparib	temozolomide	25.0
HSSY-II	AZD-1390	trabectedin	24.6
287954-098-R-J1	talazoparib	temozolomide	24.5
NCI-H841	AZD-1390	topotecan	24.3
ASPS-1	AZD-1390	trabectedin	24.1
349418-098-R	talazoparib	temozolomide	23.7
NCI-H841	AZD-1390	trabectedin	23.1
SYO-1	olaparib	temozolomide	22.3
HSSY-II	talazoparib	temozolomide	21.8
SYO-1	AZD-1390	topotecan	21.3
G-401	AZD-1390	trabectedin	21.1
HSSY-II	olaparib	temozolomide	21.1
349418-098-R	AZD-1390	topotecan	20.8
G-401	talazoparib	temozolomide	20.5
ASPS-1	AZD-1390	topotecan	20.5
SW 982	nedisertib	topotecan	19.9
G-401	AZD-1390	topotecan	18.4
VA-ES-BJ	AZD-1390	topotecan	18.4
287954-098-R-J1	olaparib	trabectedin	17.4
DMS 114	AZD-1390	topotecan	16.7
COR L88	elimusertib	topotecan	16.7
COR L88	AZD-1390	topotecan	16.6
G-401	olaparib	temozolomide	16.4
ASPS-1	nedisertib	trabectedin	16.2
287954-098-R-J1	elimusertib	trabectedin	15.4
DMS 114	talazoparib	topotecan	15.2
