# Supplementary material for: Multicellular Complex Tumor Spheroid Response to DNA Repair Inhibitors in Combination with DNA-damaging Drugs
Source: Cancer Res Commun. 2023 Aug 25;3(8):1648–61. doi: 10.1158/2767-9764.CRC-23-0193 (PMC10452929; doi:10.1158/2767-9764.CRC-23-0193)
Supplement: Supplementary Table 2 — Table S2. Links to the PubChem assays. [file crc-23-0193-s07.pdf]

Supplementary Table S2. Links to the PubChem assays

Name	AID	Link to access data
Anticancer human tumor 156681-154-R-J1 cell line growth inhibition	1845195	https://pubchem.ncbi.nlm.nih.gov/bioassay/1845195
Anticancer human tumor 283228-195-R-J1 cell line growth inhibition	1845193	https://pubchem.ncbi.nlm.nih.gov/bioassay/1845193
Anticancer human tumor 287954-098-R-J1 cell line growth inhibition	1845194	https://pubchem.ncbi.nlm.nih.gov/bioassay/1845194
Anticancer human tumor 292921-168-R cell line growth inhibition	1845196	https://pubchem.ncbi.nlm.nih.gov/bioassay/1845196
Anticancer human tumor 349418-098-R cell line growth inhibition	1845197	https://pubchem.ncbi.nlm.nih.gov/bioassay/1845197
Anticancer human tumor 425362-245-T-J1 cell line growth inhibition	1845198	https://pubchem.ncbi.nlm.nih.gov/bioassay/1845198
Anticancer human tumor 556581-035-R-J1 cell line growth inhibition	1845199	https://pubchem.ncbi.nlm.nih.gov/bioassay/1845199
Anticancer human tumor ASPS-1 cell line growth inhibition	1845200	https://pubchem.ncbi.nlm.nih.gov/bioassay/1845200
Anticancer human tumor COR L88 cell line growth inhibition	1845201	https://pubchem.ncbi.nlm.nih.gov/bioassay/1845201
Anticancer human tumor DMS 114 cell line growth inhibition	1845202	https://pubchem.ncbi.nlm.nih.gov/bioassay/1845202
Anticancer human tumor G-401 cell line growth inhibition	1845203	https://pubchem.ncbi.nlm.nih.gov/bioassay/1845203
Anticancer human tumor HSSY-II cell line growth inhibition	1845204	https://pubchem.ncbi.nlm.nih.gov/bioassay/1845204
Anticancer human tumor NCI-H1618 cell line growth inhibition	1845206	https://pubchem.ncbi.nlm.nih.gov/bioassay/1845206
Anticancer human tumor NCI-H1876 cell line growth inhibition	1845207	https://pubchem.ncbi.nlm.nih.gov/bioassay/1845207
Anticancer human tumor NCI-H196 cell line growth inhibition	1845208	https://pubchem.ncbi.nlm.nih.gov/bioassay/1845208
Anticancer human tumor NCI-H211 cell line growth inhibition	1845209	https://pubchem.ncbi.nlm.nih.gov/bioassay/1845209
Anticancer human tumor NCI-H226 cell line growth inhibition	1845210	https://pubchem.ncbi.nlm.nih.gov/bioassay/1845210
Anticancer human tumor NCI-H322M cell line growth inhibition	1845211	https://pubchem.ncbi.nlm.nih.gov/bioassay/1845211
Anticancer human tumor NCI-H719 cell line growth inhibition	1845212	https://pubchem.ncbi.nlm.nih.gov/bioassay/1845212
Anticancer human tumor NCI-H841 cell line growth inhibition	1845213	https://pubchem.ncbi.nlm.nih.gov/bioassay/1845213
Anticancer human tumor PDX-BL-0293-F563 cell line growth inhibition	1845218	https://pubchem.ncbi.nlm.nih.gov/bioassay/1845218
Anticancer human tumor ST8814 cell line growth inhibition	1845214	https://pubchem.ncbi.nlm.nih.gov/bioassay/1845214
Anticancer human tumor SW 1271 cell line growth inhibition	1845215	https://pubchem.ncbi.nlm.nih.gov/bioassay/1845215
Anticancer human tumor SW 982 cell line growth inhibition	1845216	https://pubchem.ncbi.nlm.nih.gov/bioassay/1845216
Anticancer human tumor SYO-1 cell line growth inhibition	1845219	https://pubchem.ncbi.nlm.nih.gov/bioassay/1845219
Anticancer human tumor VA-ES-BJ cell line growth inhibition	1845217	https://pubchem.ncbi.nlm.nih.gov/bioassay/1845217
